# Talcum powder or aqueous gel to aid external cephalic version: a randomised controlled trial

**DOI:** 10.1186/1471-2393-14-49

**Published:** 2014-01-28

**Authors:** Narayanan Vallikkannu, Wan Nordin Nadzratulaiman, Siti Zawiah Omar, Khaing Si Lay, Peng Chiong Tan

**Affiliations:** 1 Department of Obstetrics and Gynaecology, Faculty of Medicine, University of Malaya, Lembah Pantai, Kuala Lumpur 50603, Malaysia

**Keywords:** External cephalic version, Aqueous gel, Talcum powder, Randomised trial

## Abstract

**Background:**

External cephalic version (ECV) is offered to reduce the number of Caesarean delivery indicated by breech presentation which occurs in 3-4% of term pregnancies. ECV is commonly performed aided by the application of aqueous gel or talcum powder to the maternal abdomen. We sought to compare gel with powder during ECV on achieving successful version and increasing tolerability.

**Method:**

We enrolled 95 women (≥ 36 weeks gestation) on their attendance for planned ECV. All participants received terbutaline tocolysis. Regional anaesthesia was not used. ECV was performed in the standard fashion after the application of the allocated aid. If the first round (maximum of 2 attempts) of ECV failed, crossover to the opposing aid was permitted.

**Results:**

48 women were randomised to powder and 47 to gel. Self-reported procedure related median [interquartile range] pain scores (using a 10-point visual numerical rating scale VNRS; low score more pain) were 6 [5-9] vs. 8 [7-9] P = 0.03 in favor of gel. ECV was successful in 21/48 (43.8%) vs. 26/47 (55.3%) RR 0.6 95% CI 0.3-1.4 P = 0.3 for powder and gel arms respectively. Crossover to the opposing aid and a second round of ECV was performed in 13/27 (48.1%) following initial failure with powder and 4/21 (19%) after failure with gel (RR 3.9 95% CI 1.0-15 P = 0.07). ECV success rate was 5/13 (38.5%) vs. 1/4 (25%) P = 0.99 after crossover use of gel or powder respectively. Operators reported higher satisfaction score with the use of gel (high score, greater satisfaction) VNRS scores 6 [4.25-8] vs 8 [7-9] P = 0.01.

**Conclusion:**

Women find gel use to be associated with less pain. The ECV success rate is not significantly different.

**Trial registration:**

The trial is registered with ISRCTN (identifier ISRCTN87231556).

## Background

The singleton fetus presents by the breech in 3-4% of pregnancies at term [1]. The Term Breech Trial reported in 2000 that perinatal mortality, neonatal mortality, or serious neonatal morbidity was significantly lower for the planned Caesarean section group than for the planned vaginal birth group without any differences in maternal mortality or serious maternal morbidity [2]. The trial report has led to a rapid change in guideline [3], opinion [4] and actual practice across the globe [5,6]. More recently, guidelines have suggested a trial of breech vaginal birth under a strict protocol in well informed women is acceptable [7,8].

Following the Term Breech Trial report, a survey of Canadian providers reported 89% offer external cephalic version (ECV) as a means of avoiding Caesarean delivery for breech presentation [9]. Attempting ECV at term reduces the chance of non-cephalic births and of Caesarean delivery [10]. In a recent literature review comprising 84 studies reporting on 12955 version, the pooled complication rate of ECV was 6.1% (mostly transient fetal heart rate abnormalities), 0.24% for serious complications and 0.35% for emergency cesarean deliveries with the conclusion that ECV is a safe procedure [11]. An ECV trial can be cost-effective when compared to a scheduled Caesarean for breech presentation provided the probability of successful ECV is > 32% [12]. Tocolysis and regional anaesthesia improve the success rate of ECV [13].

Patient characteristics also influence ECV success. Multiparity (odds ratio [OR] 2.5; 95% confidence interval [CI] 2.3-2.8) and lower maternal weight (< 65 kg; OR 1.8; 95% CI, 1.2-2.6) are clinical predictors for successful ECV [14]. Posterior placenta location (OR 1.9; 95% CI 1.5-2.4), higher amniotic fluid index >10 (OR, 1.8; 95% CI 1.5-2.1) and flexed breech position (OR 2.3; 95% CI 1.9-2.8) are ultrasound predictors of successful ECV [15].

Talcum powder or gel is in common use in Malaysia [16] and Australia [17] to aid ECV. Talcum powder was originally used when ECV was reintroduced as a routine clinical service in our centre. Recently some of our providers have started using aqueous gel. The relative tolerability and merit of powder or gel in aiding ECV is not known. We performed a non-inferiority randomised trial to compare powder against aqueous gel, powered on self-reported procedure related maternal pain.

## Methods

The trial was conducted in a university hospital delivering 6-7000 women a year in Kuala Lumpur, Malaysia. Ethics oversight was provided by the University Malaya Medical Center Medical Ethics Committee (approval reference no. 818.5 dated 20 October 2010). An internal grant was provided by the University of Malaya (grant reference RG370/11HTM) for the running of the trial. The trial is registered with ISRCTN (identifier ISRCTN87231556) and complied with the Declaration of Helsinki.

Providers approached potential participants as they attended for their ECV appointment. A Patient Information Sheet was provided and further query was fielded by the enrolling provider. Written consent was obtained from all who agreed to participate. In our centre, ECV is performed by providers on duty in the delivery suite who could be registrars or specialists. All had performed ECVs previously but registrars were generally less experienced.

Inclusion criteria were scheduled ECV, breech presentation or transverse lie, singleton gestation, gestational age ≥ 36 weeks, intact membranes, non-anomalous fetus and reassuring fetal status on cardiotocogram. Women were excluded if regular contractions were present, estimated fetal weight < 2 kg, oligohydramnios (amniotic fluid index < 5 cm), severe hypertension, recent antepartum haemorrhage, uterine scar, related allergy and any potential contraindication to vaginal delivery.

In our centre, ECV was performed within the Delivery Suite. Women scheduled for ECV were instructed to be fasted for six hours prior to their appointment. ECV was typically scheduled for 36 to 38 weeks gestation. Prior to their ECV attempt, all participants had a bedside ultrasound assessment and a cardiotocogram. 250 mcg terbutaline was administered subcutaneously 5-10 minutes prior to attempting ECV. Regional anaesthesia was not offered.

A participant was randomly allocated to “Powder” or “Gel” by the sequential opening of the lowest numbered sealed opaque envelope remaining just before the start of ECV. Randomisation was on a one–to-one ratio. The randomisation envelopes were prepared by an author (NV who was not involved in recruitment) in a single block of 100 using a computer generated randomisation sequence obtained from http://www.random.org. The numbered envelopes were prepared en-bloc at the beginning of the study and arranged in sequence in a small box in the Delivery Suite for providers to extract and open to reveal the allocated intervention. Blinding of providers and patients to the intervention was not attempted as it was considered unachievable. We used commercially available baby talcum powder and ultrasound aqueous gel.

After appropriate positioning of the participant for ECV, powder or gel was applied to the woman’s abdomen by the operator. ECV was then carried out in a standard fashion as previously described [18]. In the first round, a maximum of 2 attempts at ECV were permitted. An attempt comprised of a continuous maneuver typically lasting not more than 2-3 minutes. Fetal presentation and heart rate were then checked by ultrasound. If ECV was unsuccessful but the fetal heart rate was normal and the woman was agreeable, a second attempt was made with the same allocated aid. After completion of the first round of a maximum of two attempts, the participant was asked to record her ECV related pain score and the operator asked to provide a satisfaction score with the use of the allocated aid, using a 10 point visual numerical rating scale (VNRS - scored from 1 to 10, marked as higher score more desirable result).

Following an unsuccessful first round of ECV if the fetal status was reassuring on cardiotocogram (i.e. until at least two fetal heart rate accelerations were observed in the context of a normal baseline, baseline variability and the absence of decelerations) and the provider and woman were both willing, a second round of up to two ECV attempts was permitted; with crossover to the opposing aid i.e. powder to gel, gel to powder. A further terbutaline dose was given for the second round which was conducted in similar fashion to the first round.

Finally, whether ECV was eventually successful or otherwise, a post ECV cardiotocogram was done for all participants to obtain a reassuring trace or until intervention for an abnormal trace. If ECV was successful, unless there was a specific indication for immediate labour induction, the participants were allowed home to await spontaneous labour. If ECV was unsuccessful, women were also allowed home unless there was a specific indication for delivery, in which case Caesarean delivery was offered on the same or the next day. We did not prohibit a repeat ECV attempt on a future occasion in our protocol. Our centre operated a policy of recommending a planned Caesarean delivery (at ≥ 39 weeks gestation) for a viable singleton breech fetus.

Pregnancy outcome data were extracted from the relevant clinical charts and other hospital records after participants had been discharged following delivery and transcribed onto a standardised case report form.

We predefined self-reported ECV related VNRS pain score as primary outcome. The pain VNRS used was a horizontal line with 10-points marked at regular intervals from 1 to 10. Written instruction on the scale indicated that low score is for worse and high score is for better outcome. Participants were instructed to circle a number on the line to indicate their procedure related pain score.

ECV was considered a success if cephalic presentation was demonstrated on ultrasound immediately after an attempt. Secondary outcomes collected include operator’s VNRS satisfaction score (identical scale to the pain VNRS described above) with the agent used, significant post ECV cardiotocogram anomaly, cephalic presentation at birth, Caesarean delivery (and indication), neonatal outcomes of Apgar score, umbilical cord arterial blood pH and base deficit and neonatal admission, gestational age at delivery, blood loss at delivery and birth weight.

A previous study comparing powder and gel during ECV was not available to provide pilot data for sample size calculation. We took a 1-point increase on the 10-point VNRS for pain as non-inferior for the gel compared to powder. We assume the standard deviation of VNRS pain score to be 1.5. Taking alpha 0.05 and beta 0.1, applying the Student t test, at least 78 participants were required for a suitably powered study (calculated on http://www.sealedenvelope.com/power/continuous-noninferior/). We subsequently increased sample size by 15% after factoring in the possibility that VNRS pain score may not be normally distributed and the Mann Whitney U test will need to be applied in place of the t test resulting in a calculated sample size of 90 participants. We prepared 100 randomisation envelopes for enrollment in the event of drop outs, unforeseen post randomisation exclusions and other errors.

For assessment of major harms of the study, we looked at procedure related Caesarean delivery, fetal or neonatal death, neonatal hypoxic-ischaemic encephalopathy and major abruptio placenta.

Data was entered into SPSS 17 (SPSS Inc., Chicago, IL). Primary analysis was planned to be per protocol if there were protocol violations as is appropriate for a non-inferiority hypothesis to minimise the mistaken rejection of the null hypothesis of a significant difference between the interventions compared to intention to treat analysis [19]. The normality of distribution of continuous variables (i.e. maternal age, weight, height and body mass index, gestational age at recruitment, estimated fetal weight, amniotic fluid index, maternal pain VNRS score, provider satisfaction VNRS score, gestation at delivery, estimated blood loss at delivery, birth weight, Apgar scores and umbilical arterial blood pH and base deficit) was checked with the 1-sample Kolmogorov-Smirnov test. Normally distributed data was expressed in mean ± standard deviation and non-normally distributed or ordinal data as median [interquartile range]. The Student t test was applied in the analyses of normally distributed continuous variables (i.e, maternal age, weight, height, body mass index, estimated fetal weight, amniotic fluid index, gestation at delivery, birth weight and umbilical arterial blood pH and base deficit) with the Mann Whitney U test used in preference if data distribution was non-normal or ordinal in nature (i.e. gestational age at recruitment, parity, maternal pain VNRS score, provider satisfaction VNRS score, estimated blood loss at delivery and Apgar scores). To further support the robustness of our findings of significant differences, although the data were both ordinal and non-normally distributed for the maternal pain VNRS score and provider satisfaction VNRS score, we also analysed these parameters with the Student t test and additionally expressed the data in mean ± standard deviation. Two by two categorical datasets were analyzed by Fisher’s exact test (nulliparity, ECV successes at various attempts, cross-over rates, CTG abnormality after ECV, cephalic presentation at birth and neonatal admission) and larger categorical datasets by the Chi square test (ethnicity, type of breech presentation, placenta location, mode of delivery and indication for Caesarean delivery). We performed a multivariable logistic regression analysis as it was noted that several important characteristics (i.e. maternal age, multiparity, maternal weight, placenta location, type of breech and amniotic fluid index) of the participants, characteristics that could influence ECV success in the trial arms were somewhat different (though not at the 5% significance level), to control for these characteristics in a hypothesis generating exercise on the adjusted impact of powder versus gel on ECV success. All tests were 2 sided and P < 0.05 was taken as a level of significance.

## Result

**Figure 1:**
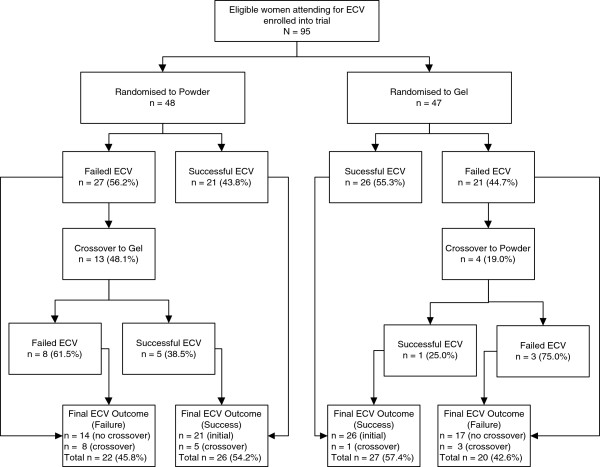
Recruitment flow chart for a randomised trial of talcum powder versus aqueous gel for external cephalic version.

The trial recruited from January 18 2011 and the last participant was delivered by December 23 2012. Figure 1 depicts the flow of trial participants including crossover to the opposing aid after initial ECV failure. Ninety five women, all with fetuses in breech presentation were enrolled and then randomized: 48 to powder and 47 to gel. Recruitment ceased when all 100 numbered envelopes were used. Five numbered envelopes could not be accounted for (two allocated to powder and three allocated to gel). ECV was performed by 37 different providers. The number of participants per provider ranged from 1 to 9. Of the provider (a registrar) who performed ECV for nine participants, five of the participants were randomised to gel and four to powder. Specialists performed ECV in 12 of the participants (six participants each was allocated to powder and gel). All participants received powder or gel as allocated for their first round of ECV, so intention to treat analysis and per protocol analysis yielded identical results. Following successful ECV, 6/53 (11.3%) reverted spontaneously to non-cephalic presentation. There was no spontaneous version to cephalic after failed ECV. With the exception of one woman who had a vaginal breech delivery, the other 47 participants with non-cephalic presentation at birth were all delivered by Caesarean. Of the 47 women with cephalic presentation who underwent trial of labour, 39 (83.0%) delivered vaginally. Although not prohibited, no repeat ECV was performed.

Table 1 depicts the characteristics of the participants stratified according to randomization to powder or gel. Participants’ characteristics across the trial arms were similar (P > 0.05).

**Table 1:** Characteristics of trial participants according to randomization to talcum powder or aqueous gel

	**Powder n = 48**	**Gel n = 47**	**P value**
	**Mean ± standard deviation, Median [interquartile range] or Number (%)**		
Age (years)	31.1 ± 4.5	29.5 ± 4.0	P = 0.07
Gestational age (weeks)	37.5 [37.4–7.9]	37.8 [37.4–38.2]	P = 0.22
Parity	1 [0–2]	0 [0–2]	P = 0.08
Nulliparous	19 (39.6)	27 (57.8)	P = 0.10
Weight (kg)	67.6 ± 9.4	71.1 ± 10.9	P = 0.10
Height (m)	1.55 ± 0.06	1.57 ± 0.06	P = 0.11
Body Mass Index	28.1 ± 4.5	28.7 ± 4.0	P = 0.50
Ethnicity			P = 0.94
Malay	35 (51.5)	33 (48.5)	
Chinese	6 (12.5)	5 (10.6)	
Indian	4 (8.3)	5 (10.6)	
Others	3 (6.3)	4 (8.5)	
Estimated fetal weight (kg)*	2.9 ± 0.3	2.9 ± 0.3	P = 0.70
Amniotic Fluid Index*	12.2 ± 3.6	11.6 ± 3.2	P = 0.43
Type of breech*			P = 0.15
Flexed	21 (43.8)	28 (59.6)	
Extended	27 (56,3)	19 (40.4)	
Placenta location*			P = 0.38
Anterior	24 (50.0)	17 (36.2)	
Posterior	21 (43.8)	27 (57.4)	
Fundal	3 (6.3)	3 (6.4)	

*Determined by ultrasound prior to ECV.

Table 2 depicts the primary outcome and ECV success outcome at each attempt stratified according to randomization to powder or gel. The primary outcome of ECV related maternal pain as expressed using a 10-point visual numerical rating scale (VNRS) was significantly worse with talcum powder compared to aqueous gel median score [interquartile range] 6 [5-9] vs. 8 [7-9] P = 0.03. First round ECV success rate between powder and gel arms was not different 21/48 (43.8%) vs. 26/47 (55.3%) RR 0.6 95% CI 0.3-1.4 P = 0.31. Including cross-overs, ECV success rates were 54.2% versus 57.4% for the powder against gel arms respectively as originally allocated. Post hoc, we considered the rate of crossovers after a failed initial round of ECV attempts. 13/27 (48.1%) who failed ECV with powder had a further round of ECV attempts with gel compared to only 4/21 (19.0%) who failed ECV with gel crossing to the use of powder for a further round of ECV, P = 0.07. We also evaluated ECV success rate taking into account all 164 ECV attempts – there were 77 attempts with powder and 87 with gel: respective success rates were 22/77 (28.6%) vs. 31/87 (35.6%) RR 0.8 95% CI 0.6-1.2 P = 0.40, again not significantly different.

**Table 2:** Primary outcome and external cephalic version (ECV) success after each attempt according to randomisation to talcum powder or aqueous gel

	**Powder n = 48**	**Gel n = 47**	**P value**	**Relative risk (95% confidence interval)**
	**Mean ± standard deviation, Median [interquartile range] or Number (%)**			
**Primary outcome**				
ECV Related Maternal Pain VNRS^*^	6 [5–9]	8 [7–9]	P = 0.03	
	6.9 ± 2.2	7.9 ± 1.7	P = 0.02	
**ECV outcomes**				
First round of ECV (up to 2 attempts) ^†^			P = 0.31	RR 0.6 (95% CI 0.3–1.4)
Successful	21 (43.8)	26 (55.3)		
Failed	27 (56.2)	21 (44.7)		
**First round of ECV (up to 2 attempts)**				
First ECV attempt			P = 0.99	RR 1.0 (95% CI 0.7–1.5)
Successful	20/48 (41.7)	19/47 (40.4)		
Failed	28/48 (58.3)	28/47 (59.6)		
Second ECV attempt (failed 1^st^ attempt) n = 56			P = 0.07	
Successful	1/28 (3.6)	7/28 (25.0)		
Failed	23/28 (82.1)	17/28 (60.7)		
Not attempted	4/28 (14.3)	4/28 (14.3)		
**Second round of ECV (up to 2 attempts)**				
Crossed-over (after failed 1^st^ round) n = 48	**To gel**	**To powder**	P = 0.07	RR 3.9 (95% CI 1.0–15)
Yes	13/27 (48.1)	4/21 (19.0)		
No	14/27 (51.9)	17/21 (81.0)		
3^rd^ ECV attempt (after cross-over) n = 17	**Using gel**	**Using powder**	P = 0.99	RR 0.8 (95% CI 0.1–6.0)
Successful	4/13 (30.8)	1/4 (25.0)		
Failed	9/13 (69.2)	3/4 (75.0)		
4^th^ ECV attempt (after failed 3^rd^ attempt) n = 12	**Using gel**	**Using powder**	P = 0.80	
Successful	1/9 (11.1)	0/3 (0.0)		
Failed	2/9 (22.2)	1/3 (33.3)		
Not attempted	6/9 (66.7)	2 /3(66.7)		
Cross-over round			P = 0.99	RR 1.9 (95% CI 0.2–23)
Successful	5/13 (38.5)	1/4 (25.0)		
Failed	8/13 (61.5)	3/4 (75.0)		
	**Powder**	**Gel**		
Final ECV outcome (cross-over included)^‡^			P = 0.84	RR 0.9 (95% CI 0.4–2.0)
Successful	26 (54.2)	27 (57.4)		
Failed	22 (45.8)	20 (42.6)		

*Pain 10 point visual numerical rating scale (VNRS) with range from 1 to 10 (lower score, more pain) self-scored by participants immediately after up to 2 attempts completed (successful or otherwise) with allocated powder or gel.

^†^Defined as cephalic presentation confirmed by ultrasound following ECV with allocated powder or gel.

^‡^Defined as cephalic presentation confirmed by ultrasound following ECV with allocated powder or gel and also after a crossover (after initial failure) to gel or powder if attempted analysed according to the original allocation to powder or gel.

Table 3 shows the other secondary outcomes stratified according to randomisation to powder or gel. Providers/operators expressed less satisfaction with the use of powder compared to gel median [interquartile range] VNRS score 6 [4.25-8] vs. 8 [7-9] P = 0.01. Other secondary outcomes of abnormal cardiotocogram after ECV needing Caesarean delivery, gestational age at delivery, cephalic presentation at birth, Cesarean delivery (and indication), blood loss at delivery and a range of neonatal outcomes were not different.

**Table 3:** Secondary outcomes according to randomisation to talcum powder or aqueous gel to aid external cephalic version (ECV)

	**Powder n = 48**	**Gel n = 47**	**P value**	**Relative risk (95% confidence interval)**
	**Mean ± standard deviation, Median [interquartile range] or Number (%)**			
**Secondary outcomes**				
Providers’ Satisfaction VNRS*	6 [4.25–8]	8 [7–9]	P = 0.01	
	6.3 ± 2.5	7.6 ± 1.9	P < 0.01	
Abnormal cardiotocogram after ECV	0 (0)	0 (0)	†	
Gestational age at delivery	39.2 ± 1.0	39.1 ± 1.1	P = 0.69	
Cephalic presentation at birth	24 (50.0)	23 (48.9)	P = 0.99	RR 1.0 (95% CI 0.7–1.5)
Mode of delivery			P = 0.94	RR 0.9 (95% CI 0.3–2.0)
Caesarean delivery	27 (56.3)	28 (59.6)	P = 0.84	
Instrumental vaginal	3 (6.3)	3 (6.4)		
Spontaneous vaginal	18 (37.5)	16 (34.0)		
Indication for Caesarean delivery			P = 0.99	
Malpresentation	22 (84.6)	24 (85.7)		
Non-reassuring fetal status	2 (7.7)	2 (7.1)		
Failure to progress in labour	2 (7.7)	2 (7.1)		
Estimated blood loss at delivery (ml)	300 [200–425]	400 [200–400]	P = 0.40	
Birth weight (kg)	3.1 ± 0.3	3.1 ± 0.3	P = 0.92	
Apgar score (1 min)	9 [9]	9 [9]	P = 0.67	
Apgar score (5 min)	10 [10]	10 [10]	P = 0.99	
Umbilical artery blood pH	7.28 ± 0.07	7.29 ± 0.09	P = 0.53	
Umbilical artery blood base deficit	3.8 ± 3.3	4.2 ± 5.3	P = 0.73	
Neonatal admission^‡^	4 (8.3)	2 (4.3)	P = 0.68	RR 2.0 (95% CI 0.4–12)

*Satisfaction 10 point visual numerical rating scale (VNRS) with range from 1 to 10 (high score, greater satisfaction) self-scored by providers attempting ECV immediately after attempt (successful or otherwise) was completed with powder or gel as originally allocated.

^†^Not calculable: two zero cells.

^‡^Of the four neonatal admissions for the powder arm (two was for transient tachypnoea of the newborn, one for a suspected cephalhaematoma following vacuum delivery and another for further observation following 1 minute Apgar of 5 with umbilical arterial cord pH of 6.99 and a base deficit of 15. A. Of the two neonatal admissions for the gel arm, one was for transient tachypnoea of the newborn and another for neonatal jaundice.

On appraising Table 1, although all characteristics on bivariate analysis had P < 0.05, there was disparity in some of characteristics e.g. age, weight, parity, type of breech and placenta location with P values close to 0.05. Post hoc, we controlled for maternal age, multiparity, maternal weight, placenta location, type of breech and amniotic fluid index in a multivariable logistic regression analysis. After adjustment, the adjusted odds ratio (AOR) was 2.1 95% CI 0.8-5.8 P = 0.14 for gel compared to powder on ECV success, a non-significant result.

There was no major unintended harm arising from the trial to our knowledge.

## Discussion

Our trial finds that ECV with gel was less painful for women, more satisfying for the operator to use but the rate of ECV success was not significantly different: our sample size was not sufficient to address the latter outcome. We find gel to be both non-inferior and superior to powder in terms of self-reported procedure related maternal pain. This is possibly due to the superior anti-abrasive effect of gel yet coupled with sufficient grip for the operator to successfully perform the ECV.

An anti-abrasive agent typically ultrasonic gel, talcum powder or mineral oil is routinely applied to the abdomen during ECV [16,17] purportedly to reduce pain, to prevent abdominal wall injury and to smooth the movement of the operator’s hands as the fetal poles are rotated to their desired position. We performed a PubMed search (via http://www.ncbi.nlm.nih.gov/pubmed) on August 12 2013 using the terms “external cephalic version powder” or “external cephalic version gel” without any limitation: no study relevant to the performance of gel and powder as an aid to ECV was identified. There seemed to be a complete lack of information on the performance of these aids.

Our data suggested that gel compared to powder may improve ECV success with AOR 2.1 95% CI 0.8-5.8 P = 0.14. However this finding is not significant and the confidence interval is wide. Meta-analyzes show that beta-mimetics may increase cephalic presentation in labour after ECV rate by OR 1.38 (95% CI 1.03-1.85) [13] and regional anaesthesia may improve ECV success rate by OR 1.58 (95% CI 1.29-1.93) [20]. Given the simplicity of gel application at ECV as an intervention and the potential magnitude of benefit as suggested by our data, further powered study is warranted. A total of 626 women (applying Fisher’s exact test) will need to be enrolled for a trial to have 80% power to detect the observed crude difference (43.8% vs. 55.3%) in ECV success rates between powder and gel. Using the first round ECV success rates of 43.8% vs. 55.3% with powder and gel respectively, a posteriori our trial had only 14.5% power to detect such a difference with alpha set at 0.05.

We noticed a trend in favour of powder to gel compared to gel to powder cross- overs after ECV failure with the originally allocated aid; 13/27 (48.1%) versus 4/21 (19.0%) P = 0.07. We did not collect information on the decision making process involved in the crossover but it seemed likely that operators had a major influence in the process as the trend appeared to favour powder to gel crossover more than gel to powder crossover in tandem with the higher satisfaction for gel use expressed by operators. This trend is counterintuitive to the finding that powder was associated with more maternal pain which one would expect to reduce powder to gel crossover if viewed from the maternal perspective.

Our trial has strengths and limitations. We analyzed per protocol as is appropriate with our non-inferiority trial design. Incomplete datasets were minimal. Our trial was properly powered a priori and power calculation indicated our trial has 80% power to detect the observed difference in procedure related maternal pain (calculator available through https://www.dssresearch.com/KnowledgeCenter/toolkitcalculators/statisticalpowercalculators.aspx). As for limitations, ideally a “placebo” (no aid) arm should be included to establish the superiority of either powder or gel as an aid to ECV over placebo. As it is we compared two commonly used aids to ECV in our setting, which in our view is ethically robust. Double blinding is impossible but we felt it is unlikely that the primary outcome we evaluated would be biased by the open design. However, it is not possible to totally exclude biases arising from interactions between providers, patients and the aid used. We found no evidence that operators tried less hard to achieve success (e.g. in foregoing second attempts or not applying adequate pressure during ECV) with a particular aid though they generally liked gel better. Cross-over to gel after failure with powder was more frequent compared to cross-over to powder from gel as the operators may have been more confident with gel. This potential bias would tend to push downstream results such as presentation at birth and Caesarean delivery rate towards null on an intention to treat basis if gel was superior. We performed a number of post hoc analyzes on ECV success per attempt with each aid and we also adjusted for characteristics that might confound: we did not find any significant difference on these (but likely underpowered) analyses. Our trial was performed by 37 providers, mostly by registrars. It is possible the ECV experience or expertise of the provider could influence the ECV success rate but specialists were not more likely to have performed ECV with a particular aid and we find no evidence amongst registrars who had performed a larger number of ECVs that allocation to a particular aid was uneven. We believe our findings to be generalisable to other populations undergoing ECV without anaesthesia as our ECV protocol followed established guideline [1].

## Conclusion

The use of aqueous gel as an aid to ECV is both non-inferior and superior to talcum powder in terms of self-reported procedure related maternal pain. ECV success rate is not significantly different but our trial was not powered to assess this outcome.

### Ethics approval

Ethical oversight is provided by the University of Malaya Medical Center Medical Ethics Committee (approval reference no. 818.5 dated 20 October 2010).

## Competing interests

The authors declare that they have no competing interests.

## Authors’ contributions

NV and WNN co-wrote the trial protocol, organised the trial, interpreted data and refined the manuscript. NV also obtained funding for the study. SZO and KSL contributed to protocol development, interpreted the data and critique the manuscript. PCT conceptualised the study, co-wrote the trial protocol, analysed and interpreted the data and drafted the manuscript. All authors read and approved the final manuscript.

## Pre-publication history

The pre-publication history for this paper can be accessed here:

http://www.biomedcentral.com/1471-2393/14/49/prepub

## References

[B1] External cephalic version and reducing the incidence of breech presentation Royal College of Obstetricians and Gynaecologists UK: Green Top Guideline No.20a December 2006 (Reviewed 2010). Accessible on http://www.rcog.org.uk/files/rcog-corp/uploaded-files/GT20aExternalCephalicVersion.pdf. Last accessed 26 May 2013 10.1111/1471-0528.14466 28299867

[B2] Hannah ME Hannah WJ Hewson SA Hodnett ED Saigal S Willan AR Planned caesarean section versus planned vaginal birth for breech presentation at term: a randomised multicentre trial. Term Breech Trial Collaborative Group Lancet 2000 356 9239 1375 1383 10.1016/S0140-6736(00)02840-3 11052579

[B3] Committee on Obstetric P ACOG committee opinion: number 265, December 2001. Mode of term single breech delivery Obstet Gynecol 2001 98 6 1189 1190 10.1016/S0029-7844(01)01708-2 11755586

[B4] Hogle KL Kilburn L Hewson S Gafni A Wall R Hannah ME Impact of the international term breech trial on clinical practice and concerns: a survey of centre collaborators J Obstet Gynaecol Can 2003 25 1 14 16 12548320 10.1016/s1701-2163(16)31077-5

[B5] Phipps H Roberts CL Nassar N Raynes-Greenow CH Peat B Hutton EK The management of breech pregnancies in Australia and New Zealand Aust N Z J Obstet Gynaecol 2003 43 4 294 297 discussion 261 10.1046/j.0004-8666.2003.00078.x 14714714

[B6] Molkenboer JF Bouckaert PX Roumen FJ Recent trends in breech delivery in the Netherlands BJOG 2003 110 10 948 951 10.1111/j.1471-0528.2003.02052.x 14550366

[B7] Practice ACoO ACOG Committee Opinion No. 340. Mode of term singleton breech delivery Obstet Gynecol 2006 108 1 235 237 10.1097/00006250-200607000-00058 16816088

[B8] Kotaska A Menticoglou S Gagnon R Farine D Basso M Bos H Delisle MF Grabowska K Hudon L Mundle W Vaginal delivery of breech presentation J Obstet Gynaecol Can 2009 31 6 557 566 567-578 19646324 10.1016/S1701-2163(16)34221-9

[B9] Hutton EK Hannah ME Barrett J Use of external cephalic version for breech pregnancy and mode of delivery for breech and twin pregnancy: a survey of Canadian practitioners J Obstet Gynaecol Can 2002 24 10 804 810 12399807 10.1016/s1701-2163(16)30473-x

[B10] Hofmeyr GJ Kulier R External cephalic version for breech presentation at term Cochrane Database Syst Rev 2012 10 CD000083 10.1002/14651858.CD000083.pub2 23076883

[B11] Grootscholten K Kok M Oei SG Mol BW van der Post JA External cephalic version-related risks: a meta-analysis Obstet Gynecol 2008 112 5 1143 1151 10.1097/AOG.0b013e31818b4ade 18978117

[B12] Tan JM Macario A Carvalho B Druzin ML El-Sayed YY Cost-effectiveness of external cephalic version for term breech presentation BMC Pregnancy Childbirth 2010 10 3 10.1186/1471-2393-10-3 20092630 PMC2826287

[B13] Cluver C Hofmeyr GJ Gyte GM Sinclair M Interventions for helping to turn term breech babies to head first presentation when using external cephalic version Cochrane Database Syst Rev 2012 1 CD000184 10.1002/14651858.CD000184.pub3 PMC4171393 22258940

[B14] Kok M Cnossen J Gravendeel L van der Post J Opmeer B Mol BW Clinical factors to predict the outcome of external cephalic version: a metaanalysis Am J Obstet Gynecol 2008 199 6 630 e631-637; discussion e631-635 18456227 10.1016/j.ajog.2008.03.008

[B15] Kok M Cnossen J Gravendeel L Van Der Post JA Mol BW Ultrasound factors to predict the outcome of external cephalic version: a meta-analysis Ultrasound Obstet Gynecol 2009 33 1 76 84 10.1002/uog.6277 19115237

[B16] Yong S External cephalic version Intl Medl J 2003 2 1 1 13 http://www.e-imj.com

[B17] Clinical Guidelines External Cephalic Version (revised April 2012). King Edward Memorial Hospital for Women Perth, Western Australia Accessible on http://www.kemh.health.wa.gov.au/development/manuals/O&G_guidelines/sectionb/2/b2.10.2.pdf. Last accessed Aug 12 2013

[B18] Collaris R Tan PC Oral nifepidine versus subcutaneous terbutaline tocolysis for external cephalic version: a double-blind randomised trial BJOG 2009 116 1 74 80 discussion 80-71 10.1111/j.1471-0528.2008.01991.x 19087079

[B19] Piaggio G Elbourne DR Altman DG Pocock SJ Evans SJ Group C Reporting of noninferiority and equivalence randomized trials: an extension of the CONSORT statement JAMA 2006 295 10 1152 1160 10.1001/jama.295.10.1152 16522836

[B20] Goetzinger KR Harper LM Tuuli MG Macones GA Colditz GA Effect of regional anesthesia on the success rate of external cephalic version: a systematic review and meta-analysis Obstet Gynecol 2011 118 5 1137 1144 10.1097/AOG.0b013e3182324583 22015882 PMC3199126

